# The Complexity of Weak Rhesus Positivity in Pregnancy: Challenges and Management—A Case Report

**DOI:** 10.5811/cpcem.47264

**Published:** 2025-09-23

**Authors:** Meghan Warner, Nicole Villa, Jordan Winebrenner, Steven Lewis, Lindsay Tjiattas-Saleski

**Affiliations:** * Edward Via College of Osteopathic Medicine - Carolinas Campus, Spartanburg, South Carolina; † Spartanburg Regional Hospital System, Department of Obstetrics and Gynecology, Spartanburg, South Carolina; ‡ Prisma Health, Department of Emergency Medicine, Greenville, South Carolina

**Keywords:** *Anti-D-immunoglobulin*, *Rh-alloimmunization*, *Rhesus Ag*, *Rh immune globulin*, *RHOgam*, *case report*

## Abstract

**Introduction:**

Determining a mother’s Rhesus (Rh) antigen status is a critical component of prenatal care, guiding the administration of Rh immunoglobulin (RhIG) to prevent Rh alloimmunization, a condition that can lead to hemolytic disease of the newborn. Hemolytic disease of the newborn is a blood disorder where the blood types of a mother and fetus are incompatible and causes hemolysis of the fetus’ erythrocytes, a major cause of fetal death. Rh immunoglobulin is commonly administered to Rh-negative (Rh−) women as a prophylactic measure. However, categorizing a patient’s Rh status is not always straightforward as individuals can exhibit weakly Rh+ or formerly Rh+ phenotypes, complicating clinical management.

**Case Report:**

We present a case of a 28-year-old gravida three para two woman whose Rh status has varied across multiple pregnancies, who presented to the emergency department (ED) with an active first trimester miscarriage requesting a dose of RhIG. Her blood typing indicated O+ status, which conflicted with her previous history of O−.

**Conclusion:**

Most women in the United States are Rh+, which eliminates the need for RhIG during pregnancy. Nevertheless, approximately 550,000 women annually are categorized as Rh−, and 16,700 of these cases may represent weak Rh positivity.^1^ Identifying weakly Rh+ individuals holds potential to reallocate scarce RhIG resources to those who require them.^2^ In this report we explore the clinical implications of weak Rh positivity, emphasizing maternal-fetal health considerations and the nuanced approach required to manage such cases effectively in the ED.

## INTRODUCTION

The rhesus (Rh) antigen system is a cornerstone of transfusion medicine and prenatal care, comprising over 50 distinct antigens. The most clinically significant antigen within this system is the RhD antigen, which determines Rh positivity or negativity. Approximately 15–17% of White individuals are Rh−, while this figure is lower, at 3–8%, among those of African and Asian descent.^4^ Rhesus alloimmunization occurs when an Rh− individual is exposed to Rh+ erythrocytes, prompting the production of anti-D antibodies. These antibodies can cross the placenta during subsequent pregnancies, targeting the fetal erythrocytes and leading to hemolytic disease of the newborn.

To mitigate this risk, the American College of Obstetricians and Gynecologists (ACOG) recommends blood typing for all pregnant women > 12 weeks’ gestation, with prophylactic administration of Rh immunoglobulin (RhIG) at 28 weeks’ gestation and again postpartum if the neonate is Rh+.^3^ Since its implementation in the 1970s, RhIG has reduced alloimmunization rates by 80–90%.^4^ However, current guidelines primarily address individuals with clear Rh+ or Rh− status, leaving a gap in management strategies for those with weak Rh positivity (also known as weak D phenotype). Adding to this complexity is Rh mosaicism, where erythrocytes express varying levels of Rh antigen, resulting in conflicting test results. This phenomenon complicates diagnosis and necessitates careful clinical evaluation.

## CASE REPORT

A 28-year-old gravida three para two woman presented to the emergency department (ED) with vaginal bleeding. The patient stated that the first day of her last menstrual period was about six weeks prior to presentation. Her beta-human chorionic gonadotropin level in the ED was 27 milli-international units per milliliter, and ultrasound showed no evidence of intrauterine or extrauterine pregnancy. These findings led to the conclusion that the bleeding she was experiencing was likely sequelae of a complete and spontaneous first trimester miscarriage. The patient’s primary concern was the administration of RhIG, given her history of receiving the treatment during her prior pregnancies. During the current visit, blood indicated O+ status which conflicted with her previous history of being O−.

A thorough review of her medical history uncovered a series of inconsistencies in blood typing results across multiple pregnancies (Table). These conflicting results called attention to the challenge of accurately determining her Rh status and the potential implications for managing subsequent pregnancies.

*CPC-EM Capsule*

What do we already know about this clinical entity?

*Weak D phenotypes can produce conflicting rhesus antigen results, complicating rhesus (Rh) immunoglobulin use in miscarriage care*.

What makes this presentation of disease reportable?

*This case highlights how inconsistent Rh typing creates real-time challenges for emergency department management of miscarriage*.

What is the major learning point?

*Emergency physicians should recognize weak D variants to guide appropriate RhIG use during miscarriage care*.

How might this improve emergency medicine practice?

*This report promotes awareness of weak D testing, supporting safe RhIG use and conserving resources in miscarriage care*.

In consultation with obstetrics and gynecology (OB/GYN), the decision was made to administer RhIG prophylactically during this visit to safeguard against future complications. The patient was discharged with instructions for follow-up testing to definitively determine her Rh phenotype.

Advanced serological testing during the patient’s fourth pregnancy classified the patient as weakly Rh+. Weak D phenotypes, found in up to 1% of White women, are characterized by reduced expression of the RhD antigen on erythrocytes. Approximately 80% of individuals with this phenotype possess genotypes unlikely to cause Rh alloimmunization, while the remaining 20% harbor genotypes that could place them at risk.^1^ Although not pursued in this case, genetic testing is available as a definitive diagnostic tool. After the patient consulted with her OB/GYN and hematology, it was decided that she continue to be managed as Rh− as a prophylactic approach.

## DISCUSSION

The management of weak Rh+ individuals during pregnancy remains controversial. While most weak D phenotypes do not necessitate RhIG, exceptions exist where alloimmunization occurs. Treating all weak Rh+ individuals as Rh− is a cautious but potentially resource-intensive approach. Literature suggests that most weak Rh+ women with common genotypes do not require RhIG.^1^ Nonetheless, rare weak D genotypes can elicit anti-D antibody production, justifying the prophylactic treatment.

An estimated 13,000 pregnant women annually in the United States are weak Rh+ but receive RhIG unnecessarily, consuming approximately 24,000 doses of RhIG.^1^ Genetic testing could refine patient management by distinguishing those who truly require RhIG; however, the cost effectiveness and logistical feasibility of universal genetic testing for weak D phenotypes is prohibitive. Moreover, the growing scarcity of RhIG underscores the urgency of optimizing its use.

Early pregnancy loss or bleeding in early pregnancy account for 2.7% (900,000) of visits annually to the ED for women of reproductive age.^6^ As the ED does not always have access to old records or records from other facilities, it is not atypical for patients with these concerns to have a new blood type drawn. Given the ongoing RhIG shortage, ensuring appropriate use of RhIG is increasingly critical to preserve limited resources through prioritization and conservation while maintaining safety.^2^ The most recent ACOG guidelines recommend forgoing Rh testing and RhIG administration in patients experiencing pregnancy loss < 12 weeks’ gestation.^3^ However, the implications extend beyond obstetrics to involve blood compatibility during a transfusion. Accurate identification of weak D phenotypes also impacts transfusion medicine, particularly as blood product shortages become more pronounced. Depending on the specific weak D phenotype, some patients can be safely classified as Rh+ and receive Rh+ blood, while others should be managed as Rh− and require Rh− blood. For example, common weak D phenotypes such as 1, 2, 3, 4.0, 4.1, and 5 are generally considered Rh(D)-positive and can be transfused with Rh+ blood without increased risk of alloimmunization. In contrast, less common weak D phenotypes, including 4.2–11 and type 15 are more likely to provoke an immune response and should be treated as Rh(D)-negative and receive Rh− blood.^7^ Further research and policy development are essential to establish evidence-based guidelines for managing weak Rh+ individuals.

## CONCLUSION

This case highlights the challenges of managing weak Rh+ patients and the broader implications for maternal-fetal health and resource allocation. In both obstetrics and emergency settings, weak Rh+ patients may require careful consideration when it comes to RhIG administration, blood transfusions, and other interventions to prevent hemolytic complications. As genetic testing becomes more accessible, it may play a pivotal role in guiding the management of weak Rh+ individuals, balancing patient safety with judicious use of RhIG. In transfusion medicine, accurate determination of Rh status is essential to prevent adverse reactions, especially during trauma or critical care. Collaborative efforts are needed to address these complexities, optimize resource allocation, and improve outcomes for patients and their families across diverse clinical settings.

## Figures and Tables

**Table:** Summary of patient’s rhesus antigen (Rh) blood type results and corresponding Rh immunoglobulin (RhIG) administration across four pregnancies, demonstrating how initial and repeat blood typing, including identification of weak D variants, guided clinical decisions to administer RhIG for alloimmunization prevention.

Gestation	Initial Blood Result	On Re-check	RhIG Administered*
Gravida 1	O−	O+ O−	+ +
Gravida 2	O−	O+	+ −
Gravida 3	O+	----	+ /
Gravida 4	Weak O+	Weak O+	+ +

* The first + symbol represents the first dose of anti-Rh immunoglobulin; second + symbol represents the second dose; the − symbol indicates no dose; / indicates not applicable.
